# Validation of the Occupational Depression Inventory in Sweden

**DOI:** 10.1186/s12889-023-16417-w

**Published:** 2023-08-08

**Authors:** Markus Jansson-Fröjmark, Farzaneh Badinlou, Tobias Lundgren, Irvin Sam Schonfeld, Renzo Bianchi

**Affiliations:** 1 Centre for Psychiatry Research, Department of Clinical Neuroscience, Karolinska Institute, and Stockholm Health Care Services, Region of Stockholm, Stockholm, Sweden; 2 Department of Psychology, The City College and the Graduate Center of the City University of New York, New York City, NY USA; 3 Department of Psychology, Norwegian University of Science and Technology (NTNU), Trondheim, Norway

**Keywords:** Anxiety, Demand-control imbalance, Effort-reward imbalance, Factor analysis, Job support, Measurement invariance, Mokken scale analysis, Work stress

## Abstract

**Background:**

The Occupational Depression Inventory (ODI) was recently devised to assess depressive symptoms that individuals specifically attribute to their work. One purpose of the ODI is to overcome limitations in current assessments of job-related distress. This study aimed to validate the Swedish version of the ODI.

**Methods:**

The study involved 365 individuals employed in Sweden. In addition to the ODI, the study included the Satisfaction with Life Scale, the Effort-Reward Imbalance Questionnaire, the Demand-Control-Support Questionnaire, the GAD-2, and the PHQ-9. We inquired into the factorial validity, dimensionality, scalability, test-score reliability, criterion validity, convergent validity, discriminant validity, and measurement invariance of the ODI.

**Results:**

Exploratory structural equation modeling bifactor analysis indicated that the ODI’s Swedish version meets the requirements for essential unidimensionality (e.g., explained common variance = 0.872). Measurement invariance held across sexes, age groups, and occupational categories. The instrument exhibited strong scalability (e.g., *H* = 0.662). The observed total scores thus accurately ranked respondents on the latent continuum underlying the scale. The ODI’s total-score reliability was high (e.g., McDonald’s ω = 0.929). Speaking to the instrument’s criterion validity, we found occupational depression to correlate, in the expected direction, with various work (e.g., job support) and nonwork (e.g., general anxiety) variables. Occupational depression showed large correlations with effort-reward imbalance (*r* = 0.613) and demand-control imbalance (*r* = 0.566) at work. Multiple regression analyses supported these associations further. As expected, we observed both a degree of convergent validity and a degree of discriminant validity when examining the ODI against the PHQ-9, an attribution-free measure of depression.

**Discussion:**

This study indicates that the ODI performs well within the Swedish context, consistent with the findings obtained in other linguistic and geographic contexts. The ODI promises to help researchers, practitioners, and public health decision-makers address job-related distress more effectively.

**Supplementary Information:**

The online version contains supplementary material available at 10.1186/s12889-023-16417-w.

## Introduction

Depression is a leading cause of disease worldwide [1]. In diagnostic systems, depression is characterized as a clinical syndrome with two central symptoms, dysphoria and anhedonia [2, 3]. Several other features are also common, such as exhaustion, feelings of guilt or worthlessness, and thoughts of suicide or suicide attempts. Community studies conducted in 30 countries estimated the one-year prevalence of depression to be 7.2% and its lifetime prevalence, 10.8% [4]. Although the diagnostic conceptualization of depression has clear clinical utility, research at the forefront of the field of psychopathology indicates that depression is best conceived of as a dimensional phenomenon, on a continuum from normal mood states to chronic, severe depressive disorders [5–7].

In the nomenclature of the American Psychiatric Association and World Health Organization, diagnoses of depression are not contingent upon specific etiological pathways and multiple causal factors can be envisaged [2, 3]. Among the factors involved in depression’s etiology, work stress has elicited considerable interest in recent years [8, 9]. It is in this context that the Occupational Depression Inventory (ODI) was developed [10]. The ODI was designed to assess the severity of work-attributed depressive symptoms and establish provisional diagnoses of job-ascribed depression. The instrument thus adopts an approach to work-attributed depressive symptoms that is both dimensional and categorical. The ODI is meant to help investigators (a) identify individuals with work-related depressive conditions and (b) estimate the prevalence of work-related depressive conditions. All ODI items focus on depressive symptoms that respondents causally attribute to their work (e.g., “My experience at work made me feel like a failure”). The ODI comprises nine core items, each reflecting one of the nine symptom criteria for a diagnosis of major depression as per the *Diagnostic and Statistical Manual of Mental Disorders*, fifth edition, text revision (DSM-5-TR) [2], and a subsidiary item evaluating turnover intention. Respondents complete the nine core items by indicating the severity of symptoms experienced over the past two weeks, consistent with DSM-5-TR’s diagnostic criteria for major depression [2, 10].

To date, the ODI has been validated in seven different countries—Australia, France, New Zealand, South Africa, Spain, Switzerland, and the USA—and in three different languages—English, French, and Spanish. Previous ODI studies have found the instrument to exhibit robust psychometric and structural properties [10–14]. Examined within an exploratory structural equation modeling (ESEM) bifactor analytic framework [15, 16], the ODI has been found to meet the requirements for essential unidimensionality. In addition, excellent test-score reliability has been observed [11, 13, 14, 17]. There is also suggestive evidence that the ODI behaves equivalently across a variety of groups (e.g., male versus female individuals, younger versus older individuals), thus allowing for meaningful comparisons between these groups. Several studies have documented the convergent and discriminant validity of the ODI vis-à-vis attribution-free (or cause-neutral) measures of depressive symptoms [10, 11, 14]. A combination of convergent and discriminant validity was found in these studies, consistent with the ODI’s focus on depressive symptoms that are specifically attributed to work.

Furthermore, the ODI has demonstrated criterion validity in relation to measures of work (job incivility, work engagement, work overload) and nonwork (e.g., general health status, objective cognitive performance) variables [10, 11, 14, 18, 19]. Speaking to the usefulness of the ODI at supra-individual levels of resolution, a recent study found occupational depression to be (a) negatively linked to companies’ stock growth and (b) positively linked to states’ economic deprivation [20]. Finally, the ODI helped address the long-debated issue of burnout-depression overlap [17, 21]. Thus far, the ODI has not been employed and validated in Nordic countries.

The present study examined the structural and psychometric properties of the Swedish version of the ODI. More specifically, we inquired into the factorial validity, dimensionality, scalability, test-score reliability, criterion validity, convergent/discriminant validity, and measurement invariance of the instrument. Based on past ODI research [10, 11, 13, 14], we expected the instrument to exhibit (a) high factorial validity and essential unidimensionality, (b) strong scalability, (c) good total-score reliability, (d) criterion validity in relation to both work and nonwork variables, (e) a degree of convergent validity and a degree of discriminant validity vis-à-vis an attribution-free (cause-neutral) depression scale, and (f) measurement invariance across sexes, age groups, and occupational categories.

We investigated the criterion validity of the ODI in relation to effort and reward at work, job-related demand, control, and support, satisfaction with life, and general anxiety. The Effort-Reward Imbalance and Demand-Control(-Support) models have been prominent in research on employee health and well-being [22–26]. We hypothesized that the ODI would show (a) negative associations with work-related reward, job control, job support, and satisfaction with life, and (b) positive associations with work-related effort, job demand, distress (as indexed by effort-reward imbalance and demand-control imbalance), and general anxiety. We investigated the convergent and discriminant validity of the ODI against the PHQ-9, a well-established, attribution-free depression scale [5, 27]. Both the ODI and the PHQ-9 are centered on the nine core symptoms of major depression. However, by design, the ODI assesses these symptoms in relation to work—in contrast to the cause-neutral content of the PHQ-9. On these bases, we expected a balance of convergent and discriminant validity when examining the ODI against the PHQ-9.

Validating the ODI in Sweden is an important step in making the instrument available to occupational health specialists based in Nordic countries. From a more global perspective, the present study has the potential to inform us further about the characteristics and nomological network of the ODI. Many authors have emphasized the need for (more) comprehensive examinations of the instruments employed in applied psychology. For instance, Cortina et al. [28] noted that “[t]he distance between actual and recommended scale development and evaluation practices may have reached a magnitude that should lead us to question our conclusions regarding organizational phenomena…” (p. 1352). It is not uncommon for researchers to find out that measures that have been in use for years (even decades) exhibit psychometric and structural flaws [29]. To avoid such problematic situations, it is essential to submit recently developed instruments to close inspection.

## Methods

### Study sample and data collection

Study participants were recruited via convenience sampling. A recruitment announcement that included information about the study and a link to a web survey was posted in occupationally-oriented Facebook groups (e.g., for nurses, teachers, and engineers), and on the Karolinska Institute homepage. The participants could answer the survey on the online platform, Research Electronic Data Capture (REDCap), hosted locally at the Karolinska Institute [30, 31]. Data were collected during February and March 2022. Inclusion criteria were: (1) working full- or part-time, (2) being at least 18 years old, and (3) reporting satisfactory reading and writing skills in the Swedish language. In total, 397 candidates responded to the web survey. Thirty-two participants had not completed all the ODI items and were therefore excluded from the study. As a result, this paper reports on the responses provided by 365 participants.

Participants’ ages ranged from 19 to 65 (*M* = 43, *SD* = 11). A large majority of the respondents were women (88.2%). Most participants had college or university as their highest level of education (79.2% college or university; 18.4% high school; 2.5% compulsory school). Approximately 3 of 4 respondents (75.9%) reported being married or having a partner (18.4% were single; 5.2% were divorced; 0.5% were widowed). About 44.9% of the participants reported having children under the age of 18; the number of children ranged from 1 to 6. A vast majority of the respondents reported Sweden as their place of birth (86.3%).

Of the participants, 71.5% reported working full-time. Respondents with part-time jobs had worked on average 29.5 h per week during the past month (range: 3–68 h). About 49.6% of the participants were employed in the healthcare sector (e.g., social worker, midwife, psychologist, nurse). For those who were employed in other sectors, common professions included teacher, administrator, and storage worker. The majority of the respondents had been employed at their present workplace for more than a year (0–1 year: 19.7%, 1–4 years: 35.3%, 4–10 years: 23.8%, and > 10 years: 21.1%). With regard to health behaviors, 8.8% of participants reported smoking; 15.8%, used snuff; and 37.8%, drank one glass of alcohol or more per week.

The study was approved by the Swedish national ethical board (DNR 2021-06120-01) and informed consent was obtained from all participants. All data collection procedures were in agreement with the ethical standards of the Helsinki Declaration of 1964 and its subsequent amendments.

### Measures of interest

#### ODI

The ODI comprises nine items referencing the symptoms of major depression found in the DSM-5-TR [2, 10]. The instrument thus assesses anhedonia, depressed mood, sleep alterations, fatigue/loss of energy, appetite alterations, feelings of worthlessness, cognitive impairment, psychomotor alterations, and suicidal ideation. As previously noted, each symptom is examined in connection to work. The symptoms are assessed within a two-week time window, based on a rating scale from 0 for “never or almost never” to 3 for “nearly every day.” The ODI includes an additional item that evaluates turnover intention (“If you have encountered at least some of the problems mentioned above, do these problems lead you to consider leaving your current job or position?”; response options: “yes,” “no,” and “I don’t know”). The ODI is associated with instructions to respondents. Respondents are invited to consider various sources for their symptoms, including work-*un*related and unknown sources, as they complete the items. Such precautions aim at discouraging hasty attributions of the experienced symptoms to work. We translated the ODI into Swedish using a back-translation method [32]. We relied on two independent translators, one translating the items from English to Swedish, and the other translating the items from Swedish to English. The items of the ODI translated into Swedish are displayed in Table 1, together with the original English items.

**Table 1:** Swedish version of the Occupational Depression Inventory (ODI)

Symptoms	Items
Anhedonia	Mitt arbete var så stressfyllt att jag inte kunde njuta av de saker som jag brukar tycka om att göra. *My work was so stressful that I could not enjoy the things that I usually like doing.*
Depressed mood	Jag kände mig nedstämd på grund av mitt arbete. *I felt depressed because of my job.*
Sleep alterations	Stressen i mitt arbete ledde till att jag fick sömnproblem (jag fick problem att somna eller att jag vaknade på natten, eller jag sov mer än vanligt). *The stress of my job caused me to have sleep problems (I had difficulties falling asleep or staying asleep, or I slept much more than usual).*
Fatigue/loss of energy	Jag kände mig utmattad av mitt arbete. *I felt exhausted because of my work.*
Appetite alterations	Jag kände att min aptit var störd på grund av stressen i mitt arbete (jag tappade aptiten, eller det motsatta, jag åt för mycket). *I felt my appetite was disturbed because of the stress of my job (I lost my appetite, or the opposite, I ate too much).*
Feelings of worthlessness	Min upplevelse på arbetet gjorde att jag kände mig som ett misslyckande. *My experience at work made me feel like a failure.*
Cognitive impairment	Mitt arbete stressade mig så mycket att jag hade svårt att fokusera på vad jag höll på med (t.ex. att läsa en tidningsartikel) eller att tänka klart (t.ex. att ta ett beslut). *My job stressed me so much that I had trouble focusing on what I was doing (e.g., reading a newspaper article) or thinking clearly (e.g., to make decisions).*
Psychomotor alterations	Som ett resultat av arbetsstress, så kände jag mig rastlös, eller motsatsen, märkbart långsam – t.ex. på det sätt som jag rörde mig eller pratade. *As a result of job stress, I felt restless, or the opposite, noticeably slowed down―for example, in the way I moved or spoke.*
Suicidal ideation	Jag tänkte att jag hellre skulle vilja vara död än att fortsätta med det här arbetet. *I thought that I’d rather be dead than continue in this job.*
Turnover intention (SQ)	Om du har stött på åtminstone några av problemen ovan, leder problemen dig att överväga att lämna ditt nuvarande arbete eller position? *If you have encountered at least some of the problems mentioned above, do these problems lead you to consider leaving your current job or position?*

The full ODI form (including the instructions to respondents) is available in Supplementary Material 1, together with an SPSS syntax implementing the provisional diagnosis algorithm of the ODI

* SQ *Subsidiary question

Based on the provisional diagnosis algorithm linked to the ODI (for a detailed description, see [10]), about 15% of the participants (*n* = 55) were identified as likely cases of occupational depression. About 54% of the participants (*n* = 196) declared that they were considering leaving their current job or position because of their depressive symptoms. Welch’s robust test of equality of means and Dunnett’s T3 indicated that these participants had higher ODI scores (*M* = 1.601, *SD* = 0.699) than (a) participants expressing no turnover intention (*M* = 0.549, *SD* = 0.564), *p* < 0.001, *d* = 1.617, and (b) undecided participants (*M* = 1.194, *SD* = 0.758), *p* < 0.001, *d* = 0.573. The full Swedish version of the ODI, including the instructions to respondents, is available in Supplementary Material 1. In addition, Supplementary Material 1 contains an SPSS syntax implementing the abovementioned algorithm. Descriptive statistics for the ODI are displayed in Table 2.

**Table 2:** Descriptive statistics for the Occupational Depression Inventory

Indicators	ODI1	ODI2	ODI3	ODI4	ODI5	ODI6	ODI7	ODI8	ODI9
Mean	1.499	1.359	1.389	1.704	1.068	1.104	1.216	1.137	0.296
Median	1	1	1	2	1	1	1	1	0
Standard deviation	1.034	1.051	1.085	1.064	1.123	1.038	1.087	1.078	0.707
Skewness (*SE* = 0.128)	0.011	0.141	0.151	-0.185	0.543	0.487	0.375	0.465	2.507
Kurtosis (*SE* = 0.255)	-1.150	-1.186	-1.259	-1.226	-1.152	-0.973	-1.162	-1.085	5.548
Minimum	0	0	0	0	0	0	0	0	0
Maximum	3	3	3	3	3	3	3	3	3

*N* = 365

*SE*  Standard error, *ODI1* Anhedonia, *ODI2* Depressed mood, *ODI3* Sleep alterations, *ODI4* Fatigue/loss of energy, *ODI5* Appetite alterations, *ODI6* Feelings of worthlessness, *ODI7* Cognitive impairment, *ODI8* Psychomotor alterations, *ODI9* Suicidal ideation

#### Satisfaction with Life Scale

Life satisfaction was assessed using the Satisfaction with Life Scale (SWLS) [33]. The SWLS comprises five items (e.g., “If I could live my life over, I would change almost nothing”) rated on a 7-point scale (from 1 for “strongly disagree” to 7 for “strongly agree”). Both McDonald’s ω and Cronbach’s α had a value of 0.906 in this study.

#### Effort-reward Imbalance Questionnaire

Effort and reward at work were assessed using the 10-item version of the Effort-Reward Imbalance Questionnaire (ERIQ) [34]. In the ERIQ, the effort subscale comprises three items (e.g., “Over the past few years, my job has become more and more demanding”) and the reward subscale comprises seven items (e.g., “I receive the respect I deserve from my superior or a respective relevant person”). Each item was rated on a scale from 1 (“strongly agree”) to 4 (“strongly disagree”). The effort subscale exhibited a McDonald’s ω of 0.801 and a Cronbach’s α of 0.799. The reward subscale exhibited a McDonald’s ω of 0.809 and a Cronbach’s α of 0.798.

Because the Effort-Reward Imbalance model assumes that distress develops when employees’ efforts outweigh their rewards, we computed the effort-to-reward ratio. Mathematically speaking, a ratio exceeding 1.000 indicates an unfavorable effort-reward imbalance.

#### Demand-control-support questionnaire

We assessed job demand, job control, and job support using the 17-item version of the Demand-Control-Support Questionnaire (DCSQ) [35]. The job demand subscale includes five items (e.g., “Do you have sufficient time for all your work tasks?”) whereas the job control and job support subscales each include six items (e.g., “Do you have the possibility to decide for yourself how to carry out your work?”; “There is good collegiality at work”). All items were rated from 1 (“never or hardly ever”) to 4 (“often”). The job demand subscale showed a McDonald’s ω of 0.805 and a Cronbach’s α of 0.802. The job control subscale showed a McDonald’s ω of 0.726 and a Cronbach’s α of 0.711. The job support subscale showed a McDonald’s ω of 0.852 and a Cronbach’s α of 0.844.

Because the Demand-Control(-Support) model assumes that job strain develops when job control is insufficient to allow job demand to be effectively dealt with, we computed the demand-to-control ratio. Mathematically speaking, a ratio exceeding 1.000 suggests an unfavorable demand-control imbalance.

#### GAD-2

We assessed general anxiety with the GAD-2 [36]. The instrument is made up of two items (“Feeling nervous, anxious, or on edge”; “Not being able to stop or control worrying”) rated on a 4-point scale (from 0 for “not at all” to 3 for “nearly every day”). Cronbach’s α was 0.874 in this study (McDonald’s ω was not computed because the scale comprises only two items).

#### PHQ-9

We employed the PHQ-9 as an attribution-free (or cause-neutral) measure of depressive symptoms [5, 27]. The PHQ-9 comprises nine items that, like the ODI’s items, cover the nine core symptoms of major depression. The items were rated from 0 (”not at all”) to 3 (”nearly every day”). McDonald’s ω was 0.905. Cronbach’s α was 0.901.

### Data analyses

We inquired into the factorial validity of the ODI using Mplus 8.7 [37]. We relied on exploratory structural equation modeling (ESEM) bifactor analysis. We treated the ODI items as ordinal and employed the weighted least squares—mean and variance adjusted—estimator. We used a target rotation, which renders the analysis confirmatory [15]. Consistent with Bianchi and Schonfeld’s [10] approach to the ODI’s structure, we considered two specific factors (or bifactors) in addition to the general factor—Occupational Depression. Two bifactors were extracted because the ODI encompasses “anhedonic-somatic” items (Items 1, 3, 4, 5, 7, and 8) and “dysphoric” items (Items 2, 6, and 9). ESEM bifactor analysis allows investigators to ascertain whether a scale that may involve a degree of multidimensionality is nevertheless “unidimensional enough” for the scale to be used based on its total score. This property is known as *essential unidimensionality* [16]. One key indicator of essential unidimensionality is the explained common variance (ECV) statistic. The ECV statistic estimates the proportion of common variance extracted that can be attributed to the general factor. At the scale level, an ECV value exceeding 0.80 is thought to support essential unidimensionality [10, 16]. In addition, we computed the ω_Hierarchical_ (ω_H_) coefficient and, based on its square root, the correlation between the general factor and the observed total scores.

We investigated the ODI’s scalability using the Mokken package version 3.0.6 [38] in R version 4.2.0 [39]. Scalability refers to the extent to which a scale’s items hierarchically align on a single dimension. The hierarchy concerns *item difficulty*. Item difficulty refers to the likelihood that an item will be endorsed by respondents. When considering a scale comprising psychopathology-related items, item difficulty relates to symptom severity [40, 41]. In the ODI, we expect, for instance, the fatigue/loss of energy item to be less “difficult” (i.e., more frequently endorsed) than the suicidal ideation item because suicidal ideation represents a much more severe symptom than fatigue/loss of energy. Scalability is indexed by *H* coefficients. As per commonly applied rules of thumb [42], scalability is weak if 0.300 ≤ *H* < 0.400, moderate if 0.400 ≤ *H* < 0.500, and strong if *H* ≥ 0.500; a scale-level *H* coefficient below 0.300 suggests that the scale of interest is not unidimensional. Pairwise *H* coefficients should be > 0; item-level *H* coefficients should be > 0.300. We computed McDonald’s ω, Cronbach’s α, Guttman’s λ-2, and the Molenaar-Sijtsma statistic to gauge the ODI’s total-score reliability.

We examined the ODI’s criterion validity based on the Pearson correlation coefficient and multiple regression analysis. We focused on the correlations of occupational depression with seven work-contextualized variables—job-related effort, job-related reward, job-related effort-reward imbalance, job demand, job control, job demand-control imbalance, and job support—and two context-free variables—satisfaction with life and general anxiety. In our multiple regression analyses, we considered job-related effort-reward imbalance, job demand-control imbalance, and job support as predictors and occupational depression, life satisfaction, and general anxiety as outcomes. Finally, we investigated the convergent and discriminant validity of the ODI vis-à-vis the PHQ-9 using the Pearson correlation coefficient and principal component analysis (PCA). PCA has proved useful in identifying between-construct overlap [43]. In our PCA, we used a promax rotation and relied on the Kaiser-Meyer-Olkin statistic to estimate sampling adequacy. According to Kaiser and Rice [44], values in the 0.800 and 0.900 s can be regarded as “meritorious” and “marvelous,” respectively (p. 112).

## Results

### Factorial validity and dimensionality

**Fig. 1:**
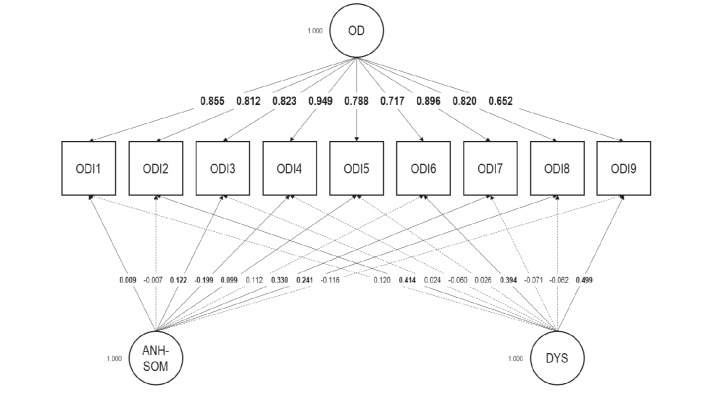
Exploratory structural equation modeling bifactor analysis of the Occupational Depression Inventory―factor loadings. Target loadings are bolded. OD: general Occupational Depression factor; ANH-SOM: Anhedonic-Somatic bifactor; DYS: Dysphoric bifactor. ODI1: anhedonia; ODI2: depressed mood; ODI3: sleep alterations; ODI4: fatigue/loss of energy; ODI5: appetite alterations; ODI6: feelings of worthlessness; ODI7: cognitive impairment; ODI8: psychomotor alterations; ODI9: suicidal ideation. *N* = 365

The bifactor model that we tested using ESEM showed a satisfactory fit: RMSEA = 0.004; CFI = 1.000; TLI = 1.000; SRMR = 0.009; *χ²* (12) = 12.073. We found all ODI items to load strongly on the general factor (0.812 on average; *SD* = 0.089), and more strongly on the general factor than on any of the two specific factors (Fig. 1). The Dysphoric bifactor was well-delineated and involved fairly substantial factor loadings ranging from 0.394 to 0.499; the Anhedonic-Somatic bifactor was weaker. With a value of 0.872, the scale-level ECV indicated that about 87% of the common variance extracted was accounted for by the general factor. Ω_H_ was 0.892, leading to a correlation between the general factor and the observed total scores of 0.945 [16]. These findings suggest that the ODI is “unidimensional enough” to be used based on its observed total scores.

Looking into the configural invariance (equivalence of the overall factor structure), metric invariance (equivalence of factor loadings), and scalar invariance (equivalence of item thresholds) of a unidimensional model, we found measurement invariance to hold across sexes (male/female), age groups (based on a median split [median age = 42]), and occupational categories (healthcare professionals/others). For each of the three variables, as we added constraints from configural to metric invariance, and from metric to scalar invariance, RMSEA never increased and CFI and TLI never decreased; SRMR never increased by more than 0.002—a very small increase [45].

### Scalability

The ODI’s scalability was strong (Table 3). The scale-level *H* coefficient reached 0.662 (95% confidence interval: 0.625, 0.700; standard error = 0.019). The pairwise *H* coefficients were well above the zero threshold. The item-level *H* coefficients largely exceeded the 0.300 threshold. The fatigue/loss of energy item (Item 4) was the least difficult item. The suicidal ideation item (Item 9) was the most difficult item.

**Table 3:** Scalability analysis of the Occupational Depression Inventory

Items	*H*_*i*_	*SE*	95% CI
ODI1 (anhedonia)	0.696	0.023	[0.651, 0.740]
ODI2 (depressed mood)	0.672	0.024	[0.625, 0.718]
ODI3 (sleep alterations)	0.665	0.026	[0.614, 0.715]
ODI4 (fatigue/loss of energy)	0.725	0.020	[0.685, 0.765]
ODI5 (appetite alterations)	0.628	0.026	[0.577, 0.680]
ODI6 (feelings of worthlessness)	0.608	0.028	[0.553, 0.663]
ODI7 (cognitive impairment)	0.703	0.021	[0.663, 0.743]
ODI8 (psychomotor alterations)	0.643	0.027	[0.591, 0.696]
ODI9 (suicidal ideation)	0.591	0.041	[0.511, 0.670]
*H*	0.662	0.019	[0.625, 0.700]

*N* = 365

*H *Scale-level *H*,  *H*_*i*_ Item-level *H,* *SE* Standard error, *95% CI* 95% Confidence interval

### Total-score reliability

The ODI’s total-score reliability was excellent. McDonald’s ω was 0.929; Cronbach’s α, 0.924; Guttman’s λ-2, 0.929; and the Molenaar-Sijtsma statistic, 0.932.

### Nomological network and criterion validity

We found statistically significant associations, in the expected direction, between occupational depression and each of our other variables of interest (see Table 4). Notably, occupational depression showed large correlations with the effort/reward ratio (*r* = 0.613, *p* < 0.001), the demand/control imbalance ratio (*r* = 0.566, *p* < 0.001), and general anxiety (*r* = 0.616, *p* < 0.001).

**Table 4:** Zero-order correlations among the main study variables

		*M*	*SD*	2.	3.	4.	5.	6.	7.	8.	9.	10.	11.
1.	Occupational depression	1.197	0.818	-0.429	0.566	-0.539	0.613	0.565	-0.325	0.566	-0.473	0.616	0.766
2.	Satisfaction with life	4.178	1.424	—	-0.161	0.389	-0.326	-0.214	0.365	-0.330	0.298	-0.444	-0.507
3.	Work-related effort	2.052	0.825		—	-0.400	0.822	0.790	-0.139	0.601	-0.231	0.340	0.356
4.	Work-related reward	2.673	0.642			—	-0.756	-0.483	0.454	-0.581	0.505	-0.362	-0.461
5.	Effort/reward imbalance	0.867	0.548				—	0.729	-0.311	0.633	-0.390	0.393	0.440
6.	Job demand	2.950	0.647					—	-0.171	0.757	-0.273	0.368	0.392
7.	Job control	2.940	0.514						—	-0.724	0.380	-0.279	-0.305
8.	Demand/control imbalance	1.047	0.349							—	-0.426	0.409	0.447
9.	Job support	3.082	0.653								—	-0.283	-0.402
10.	General anxiety	1.280	1.006									—	0.692
11.	Attribution-free depression	1.063	0.734										—

All correlations are statistically significant at *p* < 0.05 or less

We additionally note that occupational depression did not correlate with sex, *r* = 0.014, *p* = 0.791, and was only weakly associated with age, *r* = 0.111, *p* = 0.034. Occupational depression correlated positively with cigarette use, *r* = 0.148, *p* = 0.007, but negatively with alcohol consumption, *r* = -0.110, *p* = 0.044. Occupational depression did not correlate with snuff use. Welch’s analysis of variance indicated that work-attributed depressive symptoms were slightly less severe in healthcare professionals (*M* = 1.090, *SD* = 0.765) compared to other professionals (*M* = 1.303, *SD* = 0.856), *p* = 0.013, *d* = 0.262.

Our multiple regression analyses indicated that the effort/reward ratio, the demand/control ratio, and job support accounted for about 47% of the variance in occupational depression, 15% of the variance in life satisfaction, 20% of the variance in general anxiety, and 28% of the variance in attribution-free depression. All predictors were associated with the outcome variables in a statistically significant manner (see Table 5).

**Table 5:** Summary of multiple regression analyses

	Occupational depression		Life satisfaction		General anxiety		Attribution-free depression	
	*β*	*p* value	*β*	*p* value	*β*	*p* value	*β*	*p* value
Effort/reward imbalance	0.376	< 0.001	-0.150	0.029	0.191	0.004	0.203	< 0.001
Demand/control imbalance	0.229	< 0.001	-0.153	0.029	0.242	< 0.001	0.211	< 0.001
Job control	-0.236	< 0.001	0.184	0.002	-0.119	0.037	-0.253	< 0.001
Adjusted R²	0.470		0.147		0.202		0.284	

No multicollinearity issue was detected (maximum variance inflation factor = 1.790)

### Convergent and discriminant validity

On the one hand, scores on the ODI correlated 0.766 with scores on the PHQ-9, suggesting substantial convergent validity. On the other hand, results from our PCA were indicative of a degree of discriminant validity (Table 6). The PCA resulted in three interrelated components. The items of the ODI primarily loaded on the first component (Component 1). The items of the PHQ-9 primarily loaded on the second component (Component 2). The third component (Component 3) was home to the suicidality items of each scale. The emergence of a suicidality-specific component may reflect the notoriously weak endorsement of suicidality items—illustrated again in the present study. Component 1 correlated 0.650 with Component 2 and 0.433 with Component 3. Component 2 correlated 0.484 with Component 3. Although a few items showed some cross-loading (e.g., ODI’s Item 6 and PHQ-9’s Item 6), the three components were relatively well-delineated. The Kaiser-Meyer-Olkin statistic has a value of 0.921, indicating adequate sampling.

**Table 6:** Principal component analysis—pattern matrix

	Component		
	1	2	3
ODI Item 1	**0.905 **	-0.104	0.041
ODI Item 2	**0.748 **	-0.058	0.224
ODI Item 3	**0.656 **	0.284	-0.087
ODI Item 4	**0.837 **	0.057	-0.040
ODI Item 5	**0.607 **	0.241	-0.022
ODI Item 6	**0.623 **	-0.073	0.369
ODI Item 7	**0.774 **	0.190	-0.077
ODI Item 8	**0.785 **	0.141	-0.141
ODI Item 9	0.273	-0.295	**0.871 **
PHQ-9 Item 1	-0.017	**0.774 **	0.149
PHQ-9 Item 2	-0.035	**0.632 **	0.364
PHQ-9 Item 3	0.024	**0.830 **	-0.099
PHQ-9 Item 4	0.106	**0.756 **	-0.060
PHQ-9 Item 5	0.074	**0.733 **	-0.035
PHQ-9 Item 6	-0.017	**0.461 **	0.453
PHQ-9 Item 7	0.245	**0.683 **	-0.113
PHQ-9 Item 8	0.224	**0.443 **	0.084
PHQ-9 Item 9	-0.269	0.235	**0.864 **

Rotation method: promax with Kaiser normalization. The highest factor loading of each item is bolded. ODI Item 9 and PHQ-9 Item 9 both assess suicidality

*ODI* Occupational Depression Inventory

## Discussion

The ODI was recently developed to assess depressive symptoms that individuals specifically attribute to their work [10–14]. In the present study, we inquired into the psychometric and structural properties of the ODI’s Swedish version. We relied on advanced statistical analyses (e.g., ESEM bifactor analysis) allowing for close scrutiny of the instrument’s characteristics [15, 38, 45]. The need for (more) stringent examinations of instruments’ psychometric and structural properties has been recurrently underlined in applied psychology [28].

### Main findings

The Swedish version of the ODI exhibited (a) high factorial validity and essential unidimensionality, (b) strong scalability—indicating that the observed total scores accurately ranked respondents on the latent continuum underlying the scale, and (c) excellent total-score reliability. These results are consistent with the findings documented in other countries and linguistic contexts [10, 11, 13, 14]. It is of note that the ODI meets the requirements for essential unidimensionality despite covering nine different symptoms, a finding speaking to the coherence of the scale’s content. In addition, we found evidence for measurement invariance across sexes, age groups, and occupational categories, substantiating the applicability and comparability of the ODI across a broad array of individuals [45]. Our finding that fatigue/loss of energy was the most widely endorsed item and suicidal ideation was the least widely endorsed item is in keeping with past results [13], suggesting well-delineated boundaries for item difficulty.

Our study provides evidence in support of the ODI’s criterion validity. Our findings revealed large positive associations between occupational depression and both effort-reward imbalance and demand-control imbalance at work, two prominent models in research on employee health and well-being [22–26]. Occupational depression also showed substantial associations, in the expected direction, with (a) the individual components of these two types of imbalance and (b) job support, life satisfaction, and general anxiety. Our findings on the links between work-related factors and occupational depression are consistent with the ODI’s focus on depressive symptoms that individuals causally attribute to their work.

We found evidence for both a degree of convergent validity and a degree of discriminant validity when examining the ODI against the PHQ-9—an attribution-free (or cause-neutral) depression scale. This pattern of results is consistent with the view that, at the population level, only a fraction of the individuals with major depression should ascribe their disorder to job-related adversity [10]. Similar findings were obtained in past studies that examined the ODI against the 10-item version of the Center for Epidemiologic Studies Depression scale, the Depression subscale of the Hospital Anxiety and Depression Scale, and the Depression subscale of the Depression Anxiety Stress Scales-21 [10, 11, 14]. Scale differentiation manifested itself both in our PCA and in the associations of the ODI and PHQ-9 with work-related factors.

### Study limitations

This study has at least three limitations. First, we relied on a convenience sample. Although we aimed to recruit a sample reflective of the Swedish workforce, the representativeness of our sample is unclear (e.g., regarding health problems and work-related factors). While several participant characteristics were typical for Swedish citizens (e.g., concerning civil status and place of birth), our sample consisted of a majority of women and nearly 80% of the participants had completed at least some college or university education. These various concerns bear on the study’s external validity. Second, our study had a cross-sectional design, which prevented us from examining properties such as test-retest reliability or temporal measurement invariance. Carefully designed multi-wave studies are needed to clarify these questions. Third, although the value of person-reported outcome measures has been increasingly underlined in clinical research [46–49], the inclusion of objective indicators of health and performance would have been an added advantage.

### Conclusions

This study suggests that the ODI’s Swedish version has excellent psychometric and structural properties. These findings support the local use of the instrument by occupational health specialists. More globally, the present study adds to a growing body of research indicating that the ODI constitutes a robust measure of work-related depression that can help researchers, practitioners, and policymakers address job-related distress effectively.

### Supplementary Information

**Additional file 1.** Occupational Depression Inventory: SPSS syntax for a provisional diagnosis of occupational depression.

## Data Availability

The data that support the findings of this study are available on request from the corresponding author (MJF). The data are not publicly available due to that their containing information could compromise the privacy of study participants.
