# Supplementary material for: Validation of the Occupational Depression Inventory in Sweden
Source: BMC Public Health. 2023 Aug 8;23:1505. doi: 10.1186/s12889-023-16417-w (PMC10411009; doi:10.1186/s12889-023-16417-w)
Supplement: Supplementary file 1 — Additional file 1. Occupational Depression Inventory: SPSS syntax for a provisional diagnosis of occupational depression. [file 12889_2023_16417_MOESM1_ESM.docx]

**Occupational Depression Inventory: SPSS syntax for a provisional diagnosis of occupational depression**

compute DEP = 0.

do if ODI1 ge 3 or ODI2 ge 3.

count DEP = ODI3 (3)

ODI4 (3)

ODI5 (3)

ODI6 (3)

ODI7 (3)

ODI8 (3)

ODI9 (1,2,3).

end if.

if ODI1 ge 3 DEP = DEP + 1.

if ODI2 ge 3 DEP = DEP + 1.

compute DIAG = 0.

if DEP ge 5 DIAG = 1.

*Note.* The nine items of the Occupational Depression Inventory are coded ODI1 to ODI9.

Items

ODI1: anhedonia

ODI2: depressed mood

ODI3: sleep alterations

ODI4: fatigue/loss of energy

ODI5: appetite alterations

ODI6: feelings of worthlessness

ODI7: cognitive impairment

ODI8: psychomotor alterations

ODI9: suicidal ideation
